# Photooxidative stress activates a complex multigenic response integrating the phenylpropanoid pathway and ethylene, leading to lignin accumulation in apple (*Malus domestica* Borkh.) fruit

**DOI:** 10.1038/s41438-020-0244-1

**Published:** 2020-03-01

**Authors:** Carolina A. Torres, Constanza Azocar, Patricio Ramos, Ricardo Pérez-Díaz, Gloria Sepulveda, María A. Moya-León

**Affiliations:** 1 Facultad de Ciencias Agrarias, Universidad de Talca, Talca, Chile; 2 Department of Horticulture, Tree Fruit Research & Extension Center, Washington State University, Wenatchee, WA USA; 3 Universidad Andres Bello, Facultad Ciencias Biologicas, Santiago, Chile; 4 Instituto de Ciencias Biológicas, Universidad de Talca, Talca, Chile; 5 Núcleo Científico Multidisciplinario-DI, Universidad de Talca, Talca, Chile

**Keywords:** Abiotic, Light stress

## Abstract

Photooxidative stress, when combined with elevated temperatures, triggers various defense mechanisms leading to physiological, biochemical, and morphological changes in fruit tissue. Furthermore, during sun damage, apple fruit undergo textural changes characterized by high flesh firmness compared to unexposed fruit. Fuji and Royal Gala apples were suddenly exposed to sunlight on the tree and then sampled for up to 29 days. Cell wall components and lignin biosynthetic pathway analyses were carried out on the fruit tissue. At harvest, Fuji apples with different sun exposure levels, such as exposed to direct sunlight (Exp), shaded (Non-Exp), and with severe sun damage (Sev), were also characterized. In fruit suddenly exposed to sunlight, the expression levels of phenylpropanoid-related genes, phenylalanine ammonia lyase (*MdPAL*), chalcone synthase (*MdCHS*), and flavanone-3-hydroxylase (*MdF3H*), were upregulated in the skin and flesh of Exp and Sev. Exposure had little effect on the lignin-related genes caffeic acid *O*-methyltransferase 1 (*MdCOMT1*) and cinnamyl alcohol dehydrogenase (*MdCAD*) in the skin; however, the expression of these genes was highly induced in the flesh of Exp and Sev in both cultivars. Lignin deposition increased significantly in skin with sun injury (Sev); in flesh, this increase occurred late during the stress treatment. Additionally, the ethylene biosynthesis genes 1-aminocyclopropane-1-carboxylate synthase (*MdACS*) and 1-aminocyclopropane-1-carboxylate oxidase (*MdACO*) were highly expressed in the skin and flesh tissues but were more upregulated in Sev than in Exp during the time-course experiment, which paralleled the induction of the phenylpropanoid pathway and lignin accumulation. At harvest, flesh from Sev fruit exhibited higher firmness than that from Non-Exp and Exp fruit, although no differences were observed in the alcohol-insoluble residues (AIR) among groups. The fractionation of cell wall polymers revealed an increase in the uronic acid contents of the water-soluble pectin fraction (WSF) in Exp and Sev tissues compared to Non-Exp tissues, while the other pectin-rich fractions, that is, CDTA-soluble (CSF) and Na_2_CO_3_-soluble (NSF), were increased only in Sev. The amount of hemicellulose and cellulose did not differ among fruit conditions. These findings suggest that increases in the flesh firmness of apples can be promoted by photooxidative stress, which is associated with the induction of lignin accumulation in the skin and flesh of stressed fruit, with the involvement of stress phytohormones such as ethylene.

## Introduction

Photooxidative stress due to high irradiance combined with elevated temperatures during the growing season are environmental conditions that inevitably lead to photodynamic injuries of heated tissue (=sun damage) in crops growing in semiarid climates. Sun damage, commonly known as sunburn or sunscald, is a physiological disorder whose symptoms appear on the sun-exposed sections of fruit as discolorations, light and dark brown patches, bleaching, and necrosis in severe cases^1^, evidencing pigment changes (i.e., decreases in chlorophyll and increases in carotenoids) in the affected tissue^2–4^. Symptoms differ among cultivars and environmental conditions, ranging from light to dark brown spots and necrosis with or without tissue bleaching^1^. Tissue browning and necrosis on apples are developed when, in addition to high light intensities, fruit reaches a fruit surface temperature (FST) threshold of 46–49 °C and 52 ± 1 °C, respectively^1^.

This disorder triggers a plethora of metabolic responses involving the upregulation of various defense mechanisms, including antioxidant metabolites and antioxidant enzymes, causing physiological, biochemical, and morphological changes in the affected tissue^5–7^. The disorder also triggers a rapid increase in total phenolics, with particular patterns according to species and cultivar. In apples, flavonoid glycosides accumulate immediately upon exposure to stress^4,8–10^, with differences between cultivars^11^. In fact, apple tissues under different sun exposure levels exhibit significant changes in flavonoid and anthocyanin biosynthesis-related enzyme activities along with differentially accumulated metabolites^12^.

Apple tissue affected by sun damage also has increased soluble solids (mainly glucose and sorbitol), contributing to a decreased relative water content and tissue water potential, characteristics of dehydration stress^4,7,13–15^.

Other noticeable and well-documented characteristics of sun-injured tissues are their high flesh firmness compared to undamaged tissues^1,7^, perhaps due to changes in the cell and tissue anatomy characterized by compacted subepidermal layers and thick cell walls compared to unexposed tissue (unpublished data), a low relative water content^7^, and the presence of lignification and/or cell wall modifications, neither of which are fully understood in apples. In fact, on Bartlett pears, Raffo et al.^16^ reported that an increase in flesh firmness in sun-exposed fruit could be associated with a delay in pectin solubilization. Nevertheless, possible variations in the lignin content between exposed and unexposed fruit have not been investigated. In this regard, increases in lignin levels due to biotic or abiotic stresses have been associated with increases in fruit firmness in other fruit species^17,18^.

Along with the previously described metabolic and anatomical changes, sun-damaged tissue also exhibits a high ethylene concentration early in the growing season that is sustained until commercial harvest, suggesting that this phytohormone could modulate abiotic stress responses in sun-exposed fruit without triggering tissue softening, as expected^7^.

We hypothesize that the stress induction of ethylene, as well as other phytohormones, during sun injury development on apple fruit might regulate stress responses (defense and acclimation) and transduce stress signals throughout the fruit. Therefore, to gain insight into early events that lead to changes in fruit firmness during photooxidative stress, the main goal of the work was to determine the effect of the sudden exposure of apple fruit on the tree to direct sunlight on the biosynthesis and accumulation of lignin and its relationship with ethylene production. These findings will be useful for future apple breeding programs identifying the key molecular players and mechanisms that underlie the sun damage phenomena and can be used for developing new cultivars with increased fruit firmness and storability with decreased technological input.

## Results

### Fruit firmness and cell wall components in apples with different sunlight exposure levels

**Fig. 1:**
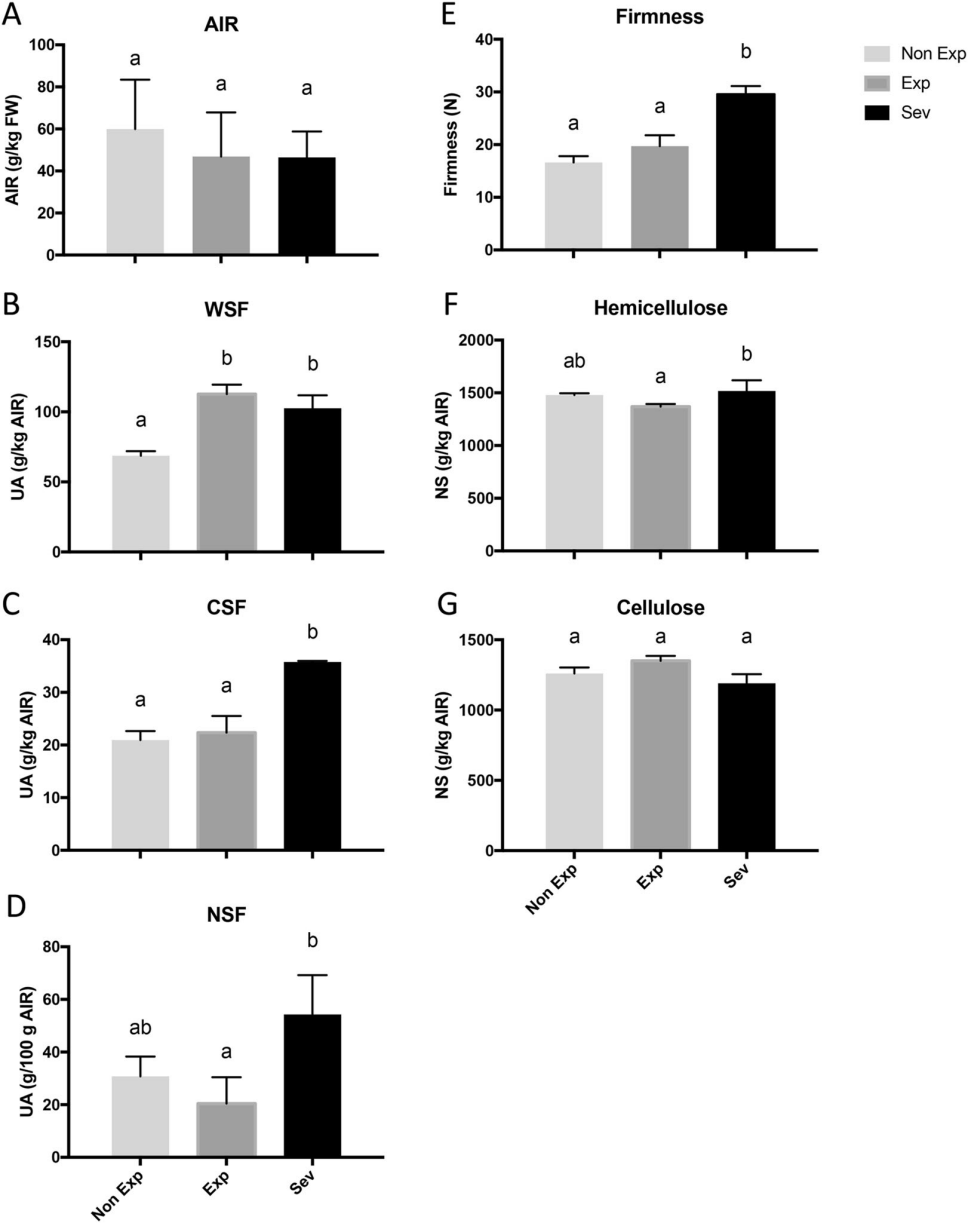
Fruit firmness and cell wall composition in apple fruit with different sunlight exposure. Alcohol-insoluble residue (AIR). (**a**) in the flesh of Fuji apples after sun exposure and the relative abundance (mean of uronic acid (UA)) of the water-soluble pectin fraction (WSF) (**b**), CDTA-soluble pectin fraction (CSF) (**c**), and Na_2_CO_3_-soluble pectin fraction (NSF) (**d**). Flesh firmness of Fuji apples grown under different sun exposure conditions at harvest time (**e**). Content (mean of neutral sugar (NS)) of hemicellulose (**f**) and cellulose (**g**) in flesh of Fuji apples after sun exposure. Non-Exp: nonexposed fruit; Exp: exposed fruit without sun damage; Sev: exposed fruit with severe sun damage. Data correspond to the mean ± SE of three biological replicates. Different letters indicate significant differences (*P* < 0.05; one-way ANOVA)

To investigate the effects of photooxidative stress caused by high solar irradiation on fruit, the contents of different cell wall polymer fractions (pectins, hemicellulose, and cellulose) were determined in Fuji apples under different levels of sunlight exposure by means of the sequential extraction of the fractions from alcohol-insoluble residue (AIR). AIR showed no changes in skin tissues from different sun exposure categories (Fig. 1a) and had an average of ~5.2%.

A two-fold increase in the proportion of the uronic acid content of the pectin-rich water-soluble fraction (WSF) (loosely bound pectin) was observed in sunlight-exposed samples (Exp and Sev) compared to Non-Exp samples (Fig. 1b). An increase in the CSF (CDTA-soluble fraction) (ionically bound pectin) was observed in severely damaged tissue (Sev) compared to sun-exposed tissue without visible damage (Exp) or non-exposed tissue (Non-Exp) (Fig. 1c). Additionally, an increase in the sodium carbonate-soluble fraction (NSF) (tightly bound pectin) was observed in Sev fruit compared to fruit from the other categories, although this difference was statistically significant only between Sev and Exp (Fig. 1d).

Additionally, the fruit firmness and cell wall composition of Fuji apples were evaluated. Tissue with severe photooxidative damage (Sev) had significantly higher flesh firmness (approximately 20%) than Non Exp or Exp tissues (Fig. 1e).

For the hemicellulose fraction, a small increase was observed in tissue with severe sun damage (Sev), although the Sev tissue was only significantly different from the Exp tissue (Fig. 1f). No changes in the cellulose content were observed among the sun exposure categories (Fig. 1g).

### Phenylpropanoid biosynthetic pathway genes in fruit during sudden exposure to direct sunlight

Because phenylpropanoids are rapidly accumulated in apple fruit tissue exposed to high solar irradiation^4,11,12^, the expression profile of phenylpropanoid biosynthetic genes leading to lignin accumulation during the development of photooxidative stress was evaluated. For this purpose, Fuji and Royal Gala apples were suddenly exposed to direct sunlight, and the expression analysis of selected genes in skin and flesh tissues was carried out over a time course experiment.

**Fig. 2:**
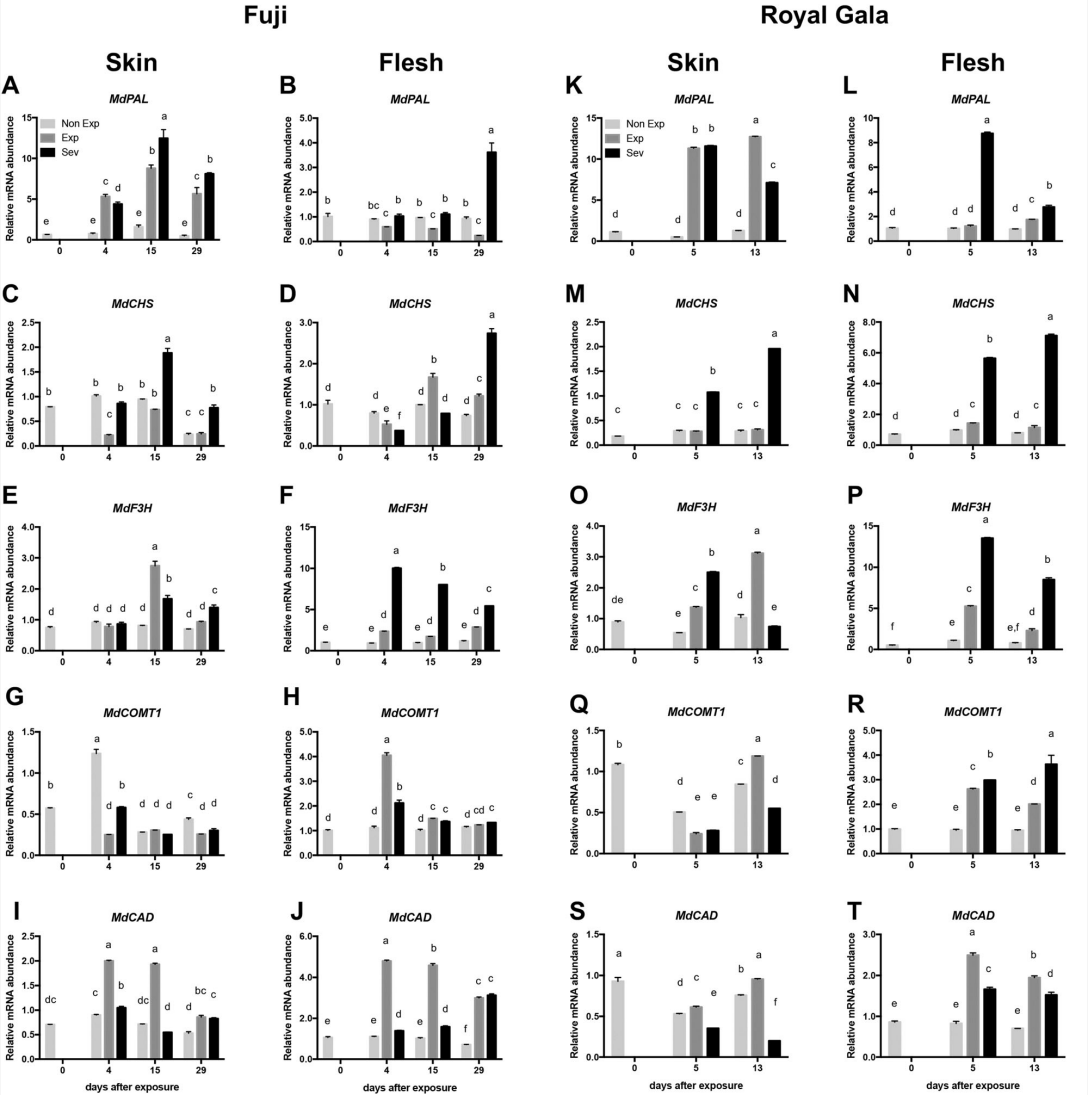
Expression analysis by RT-qPCR of phenylpropanoid biosynthesis genes in Fuji and Royal Gala apple tissues subjected to sudden sun exposure. Non-Exp: nonexposed fruit, Exp: exposed fruit without sun damage, and Sev: exposed fruit with severe sun damage. Transcript levels of *MdPAL*, *MdCHS*, *MdF3H*, *MdCOMT1*, and *MdCAD* were analyzed after different times of sun exposure in the skin (**a**, **c**, **e**, **g**, **i**, respectively) and flesh (**b**, **d**, **f**, **h**, **j**, respectively) of Fuji apples and in the skin (**k**, **m**, **o**, **q**, **s**, respectively) and flesh (**l**, **n**, **p**, **r**, **t**, respectively) of Royal Gala apples. Data correspond to the mean ± SE of three biological replicates. Different letters indicate significant differences (*P* < 0.05; two-way ANOVA)

The expression of the phenylalanine ammonia-lyase gene (*MdPAL*) was induced earlier and four-fold higher in Fuji apple skin than in flesh tissue after exposure (Fig. 2a). According to the time course experiment, the mRNA abundance of *MdPAL* increased over time in sun-exposed skin, reaching a maximum level after 15 days of exposure. Higher levels of transcripts were obtained in damaged fruit (Sev) than in exposed fruit without symptoms (Exp). Additionally, significant differences were found between tissues with severe sun damage and sun exposed tissue without sun damage at all times (Fig. 2a). In flesh samples, the expression of *MdPAL* was only induced (upregulated) in severely sun-damaged tissue after 29 days of sudden sun exposure (Fig. 2b).

The expression of flavonoid branch-related genes of the phenylpropanoid biosynthetic pathway was also determined. In skin tissues, chalcone synthase (*MdCHS*) showed a strong reduction after the exposure of the fruit to direct sunlight that recovered after 15 days (Fig. 2c). The expression of *MdCHS* increased two-fold in severely damaged tissue after 15 days of sunlight exposure (Fig. 2c). *MdCHS* in Fuji flesh exposed to direct sunlight showed a significant induction after 15 days and after 29 days in fruit with severe sun damage (Fig. 2d).

Flavonone-3 hydroxylase transcripts (*MdF3H*) showed an accumulation after 15 days in both the Exp and Sev skin tissues (Fig. 2e). In flesh, this accumulation occurred throughout the time course experiment, and it was strongly upregulated in Sev tissue (Fig. 2f).

The other branch of the phenylpropanoid pathway is monolignol biosynthesis. The caffeic acid *O*-methyl transferase 1 (*MdCOMT1*) gene expression profile was also evaluated. Interestingly, after sun exposure, *MdCOMT1* was repressed in fruit skin (Fig. 2g), while in flesh, MdCOMT1 expression was rapidly induced after 4 days of sun exposure but then recovered to normal expression levels (Fig. 2h).

Finally, the cinnamyl alcohol dehydrogenase gene (*MdCAD*) in skin was induced at all times in exposed fruit (Exp) (Fig. 2i), while in the Fuji flesh, the expression of this gene was induced in exposed fruit (Exp) in the early period and in fruit with severe sun damage (Sev) 29 days after exposure (Fig. 2j).

In Royal Gala, *MdPAL* was strongly induced after sun exposure in the skin and flesh of the suddenly exposed fruit (Exp) and fruit with severe sun damage (Sev) (Fig. 2k, l). A similar pattern was shown for *MdCHS*, with a strong induction after 5 days of sun exposure in fruit with severe sun damage (Fig. 2m, n). *MdF3H* was induced in both tissues, with a greater induction in flesh than in skin, throughout the time-course experiment (Fig. 2p, o, respectively). In the case of genes involved in lignin biosynthesis, *MdCOMT1* and *MdCAD* were mainly induced in the flesh of Royal Gala fruit in the exposed (Exp) and severe sun damage (Sev) groups compared to Non-Exp fruit (Fig. 2s, t).

### Lignin accumulation in fruit during sudden exposure to direct sunlight

**Fig. 3:**
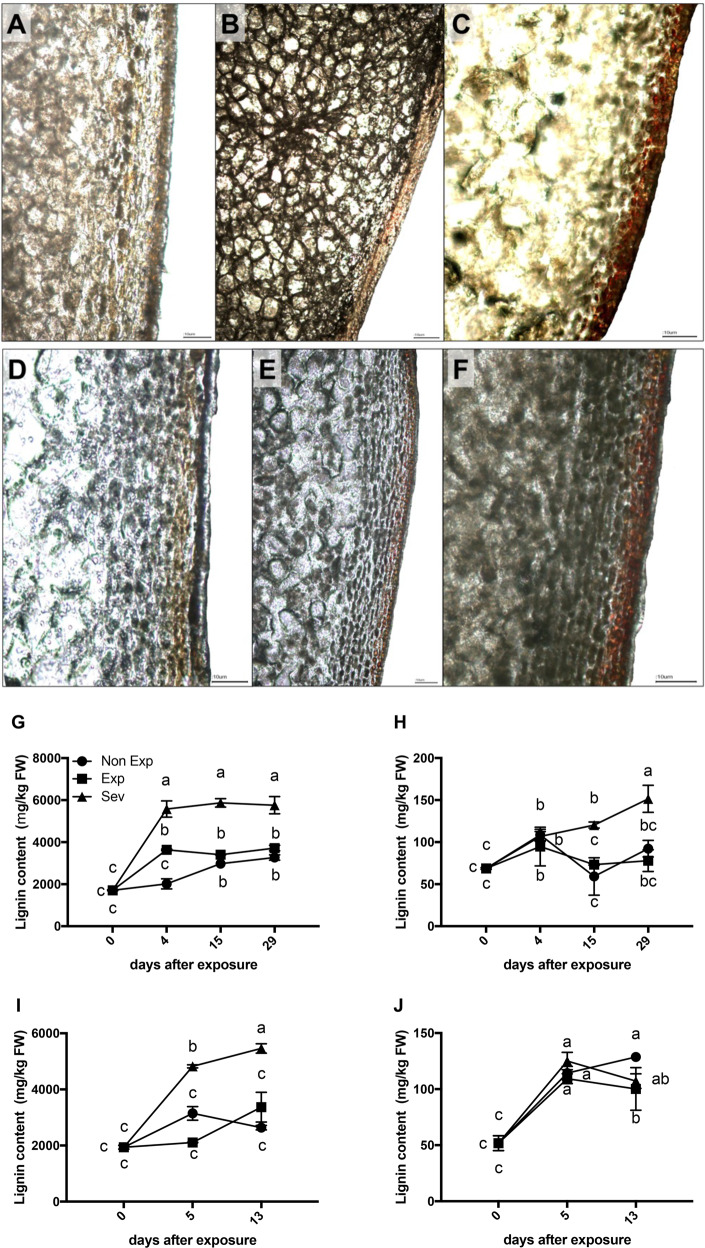
Lignin accumulation during sun damage development in apple fruit. Representative apple fruit sections after sun exposure observed under a light microscope in Fuji (**a**–**c**) and Royal Gala (**d**–**f**) apples after 160 and 120 DAFB, respectively. Non-Exp: nonexposed fruit (**a**, **d**); Exp: exposed fruit without sun damage (**b**, **e**); Sev: exposed fruit with severe sun damage (**c**, **f**). Fruit slices were stained with phloroglucinol-HCl solution (48 h) to show progressive lignin accumulation. Bars = 10 µm. Lignin content analysis in apple fruit after sun exposure. Lignin was quantified in the skin (**g**) and flesh (**h**) tissues of Fuji apples and in the skin (**i**) and flesh (**j**) tissues of Royal Gala apples. Data correspond to the mean ± SE of three biological replicates. Different letters indicate significant differences (*P* < 0.05; two-way ANOVA)

To evaluate the lignin content, an analysis was performed using microscopy and phloroglucinol-HCl lignin staining. A reddish-purple color suggested lignin accumulation, which was, in both the Fuji and Royal Gala cultivars, more intense in the skin of fruit with severe sun damage (Sev) (Fig. 3c, f, respectively) than in Non-Exp and Exp fruit (Fig. 3a, d, and b, e).

The lignin content in the tissue progressively increased in both cultivars as the time of sudden sun exposure increased (Fig. 3). In the skin tissue, lignin accumulation was significantly higher in Sev than in the other treatments in both cultivars, Fuji and Royal Gala (Fig. 3g, i, respectively). Nevertheless, only flesh from Fuji apples with Sev sun injury symptoms showed an increase (*P* ≤ 0.05) in the lignin content after 15 days (Fig. 3h). Furthermore, flesh firmness was 7% higher after 13 days of sudden exposure in Sev fruit than in Non-Exp fruit (data not shown).

### Ethylene biosynthesis genes and hormone production in fruit during sudden exposure to direct sunlight

**Fig. 4:**
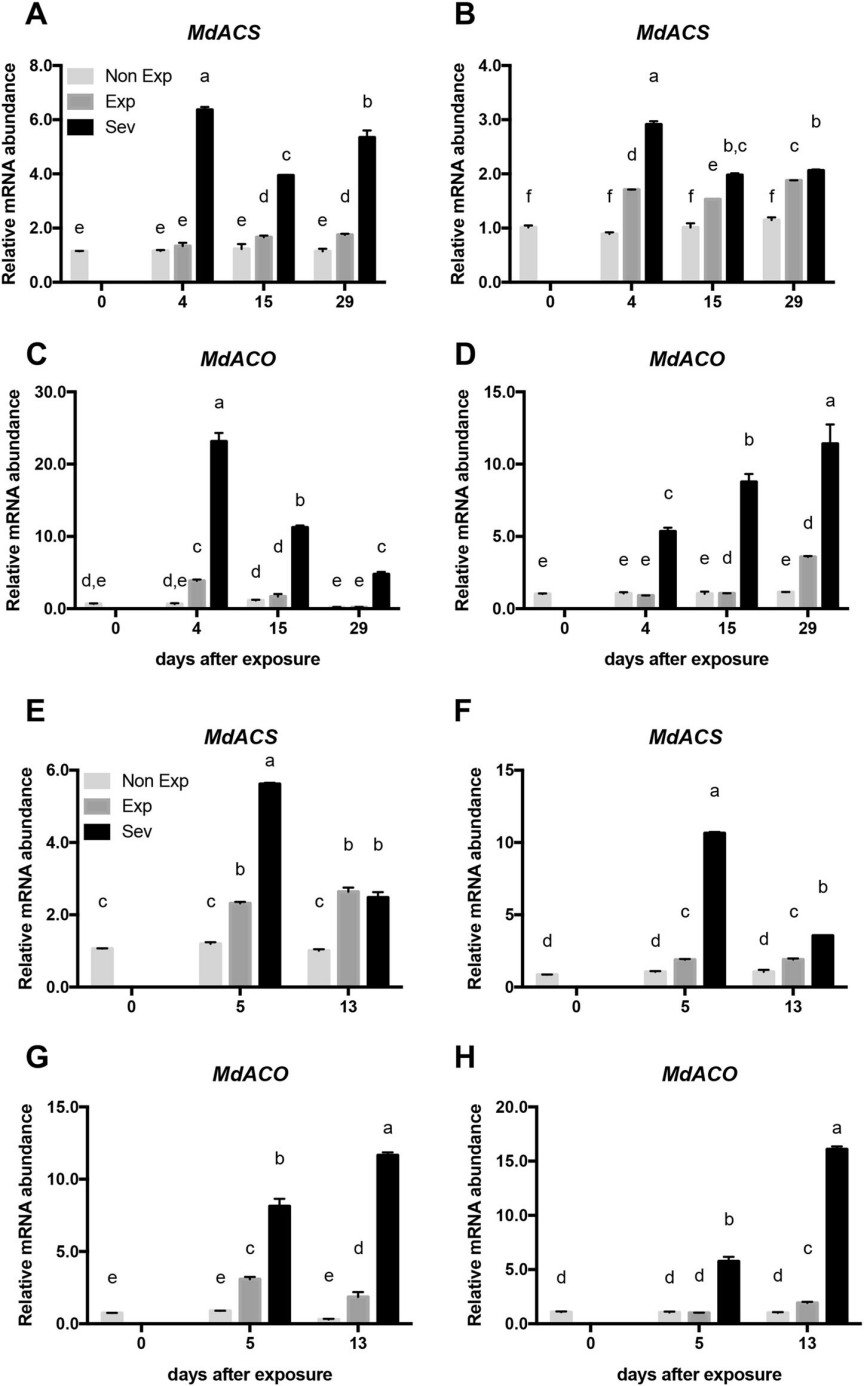
Expression analysis by RT-qPCR of ethylene biosynthesis genes in apple tissues subjected to sudden sun exposure. Transcript levels of 1-aminocyclopropane-1-carboxylate synthase (*MdACS*) and 1-aminocyclopropane-1-carboxylate oxidase (*MdACO*) were analyzed after different times of sun exposure in the skin **(a**, **c**) and flesh (**b**, **d**) of Fuji apples and in the skin (**e**, **g**) and flesh (**f**, **h**) of Royal Gala apples. Non-Exp: nonexposed fruit; Exp: exposed fruit without sun damage; Sev: exposed fruit with severe sun damage. Data correspond to the mean ± SE of three biological replicates. Different letters indicate significant differences, with Non Exp fruit as the control (*P* < 0.05; two-way ANOVA)

The expression profiles of ethylene biosynthesis genes, 1-aminocyclopropane-1-carboxylate synthase (*MdACS*) and 1-aminocyclopropane-1-carboxylate oxidase (*MdACO*), in tissues from all treatments, were analyzed. The expression of *MdACS* increased 6-fold and 3-fold in the skin and flesh tissues of Sev Fuji apples, respectively, after 4 days of sudden exposure (Fig. 4a, b). *MdACO* was also strongly induced early after sun exposure in both the skin (25-fold) and flesh tissues of Sev fruit (Fig. 4c, d). The expression of this gene displayed a maximum 12-fold increase after 29 days in the flesh of Sev fruit (Fig. 4d).

As in Fuji, in Royal Gala apples, *MdACS* was rapidly induced in the skin (6-fold) and flesh (11-fold) of Sev tissue after 5 days of sudden exposure (Fig. 4e, f). *MdACO* was strongly upregulated in the skin and flesh tissues of Sev after 5 days of sun exposure (Fig. 4g, h). The expression of *MdACO* reached a maximum level at the end of the time course in both the skin (12-fold) and flesh (15-fold) tissues (Fig. 4g, h).

**Fig. 5:**
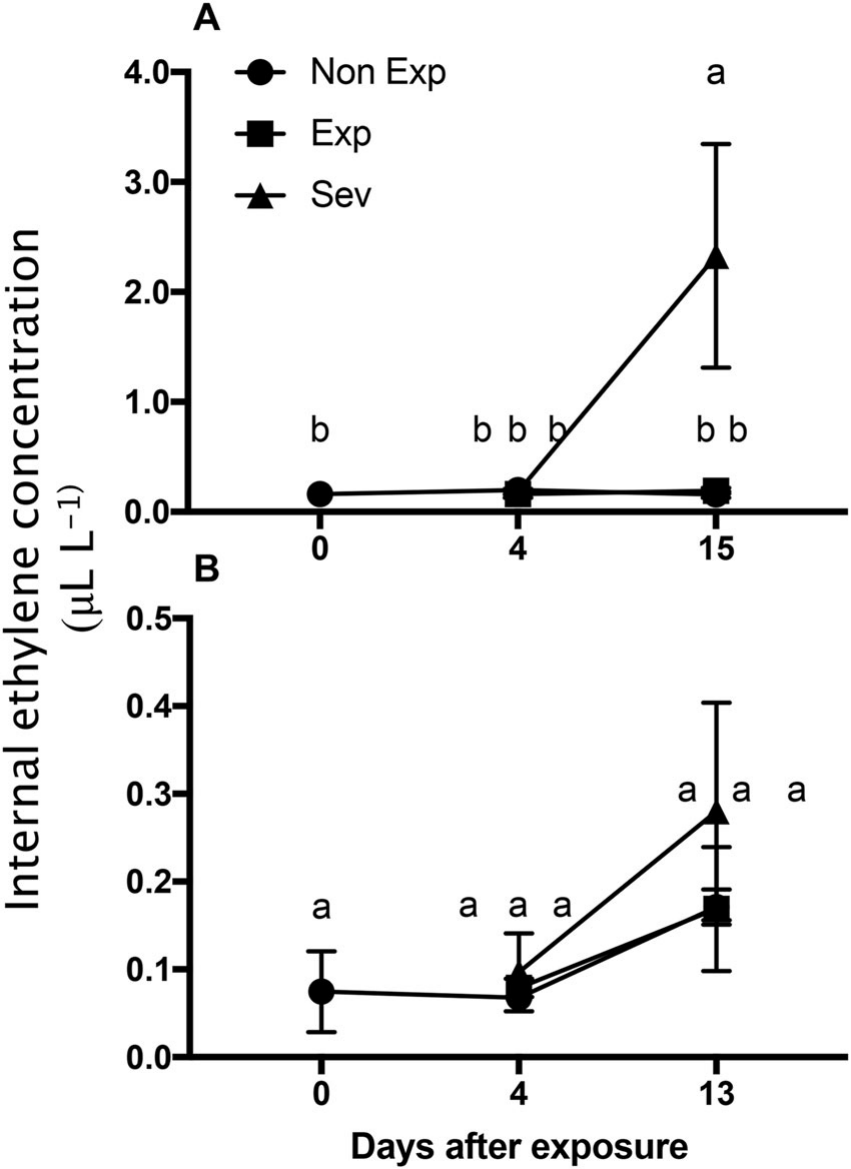
Ethylene production in apple fruit during sun damage development. Effect of photooxidative stress on the internal ethylene concentration (IEC) in Fuji (**a**) and Royal Gala (**b**) apples subjected to sudden sun exposure. Non-Exp: nonexposed fruit; Exp: exposed fruit without sun damage; Sev: exposed fruit with severe sun damage. The internal ethylene concentration (IEC) was quantified after different times of sun exposure. Data correspond to the mean ± SE of three biological replicates. Different letters indicate significant differences (*P* < 0.05; two-way ANOVA)

To evaluate the production of this hormone, as suggested by the expression pattern of key biosynthetic genes, the fruit internal ethylene concentration (IEC) was assessed. Fruit with Sev symptoms showed a moderate increase after 2 weeks of sun exposure compared with Non-Exp and Exp fruit, although this increase was only significant in Fuji apples (Fig. 5).

## Discussion

### Fruit firmness in sun-injured tissues

Changes in fruit textural properties such as flesh firmness are among the nonvisible modifications that occur in sun-damaged apple tissue. Although increased flesh firmness in the sun-injured side of apple fruit has been previously reported^7,14,15,19^, little is known concerning the changes in their cell wall structure that ultimately lead to this textural characteristic. This unique phenotype triggered by high irradiation has been described in other fleshy fruits, including pears and avocado^16,20^. In this study, we evaluated whether changes in flesh firmness in sun-exposed apple fruit can be explained by modifications in cell wall components and/or lignin accumulation.

Alcohol-insoluble residues (AIR) have been shown to decrease during fruit ripening in many species, including pears^16,21,22^. At commercial harvest, sun-injured fruit sections were firmer than nonexposed fruit sections, but this difference had no association with AIR (Fig. 1e). Similar results have been reported in sun-exposed Bartlett pears^16^.

Nonetheless, the higher levels of the WSF, CSF, and NSF pectin fractions in Exp and Sev (Fig. 1b–d) than in Non-Exp indicate Exp and Sev tissues were at a more advanced ripening stage than Non-Exp tissues. An increased ethylene concentration in sun-injured tissue could be responsible for this result^7^, but it would not explain the increased fruit firmness (Fig. 1e).

Flesh softening in apples is highly dependent upon the presence of ethylene but requires a high concentration of this hormone compared to other early ripening events, such as starch degradation (highly sensitive to ethylene but mostly ethylene-independent)^23^.

Raffo et al.^16^ found that pectin solubilization was delayed in sun-exposed Bartlett pears compared to unexposed pears during ripening after harvest, mostly as a consequence of rhamnogalacturonan-I side chain removal. During pear ripening, major changes in the noncellulosic polysaccharides of the cell walls occur^21^. In apples, as ripening progresses, cell wall soluble carbohydrates from pectin (mainly galacturonic acid) and glucose from cellulose and hemicellulose increase^24^. In our study, the cellulose and hemicellulose contents did not differ among tissues with different sun injuries (Fig. 1f, g).

A decrease in cell wall adhesion leading to changes in flesh texture has been attributed to pectin metabolism^25^. Thus, differences in pectin components observed at harvest would not explain the firmness differences in apples with different sun exposure and sun injury levels.

### Photooxidative stress in apples involves the upregulation of phenylpropanoid genes and lignin accumulation

Excess solar radiation during fruit growth is critical in sun injury development. A known defense mechanism against photooxidative stress is flavonoid accumulation, as flavonoids act as solar filters and antioxidants^26,27^. This accumulation occurs through the upregulation of the phenylpropanoid pathway, as reported for apples^4,9,10^. As shown in Fig. 2, transcriptional analysis of phenylpropanoid synthetic genes revealed the upregulation of most of the genes, with differences in their transcript abundance and behavior.

PAL is the initial enzyme in the phenylpropanoid pathway, and the apple gene encoding PAL (*MdPAL*) was highly induced in skin in response to sun exposure; this induction was generally greatest in the fruit of both cultivars that developed severe sun damage (Fig. 2a, k, respectively). In flesh tissue, this induction was delayed (Fig. 2b, l, respectively). These results are in agreement with those reported by Zhang et al.^28^ on Fuji apple skin subjected to photooxidative stress after the exposure of bagged fruit to sunlight for 3 days.

The flavonoid-related genes *MdCHS* and *MdF3H* were upregulated in both the skin and flesh of sun-injured tissue, in general, with a higher increase in tissues from Royal Gala than in tissues from Fuji (Fig. 2c, d, m, n, e, f, o, p). On the other hand, sun exposure had little effect at the transcriptional level on genes involved in monolignol biosynthesis, *MdCOMT1* and *MdCAD*, in the skin of Exp and Sev fruit, but these genes were significantly induced in the flesh of both cultivars (Fig. 2h, j, r, t). These results suggest that under photooxidative stress, the upregulation of phenylpropanoid-related genes could be a systemic response (from skin to flesh) in fleshy fruit, potentially mediated by phytohormones and/or ROS^29^.

ROS have been found to act as primary signal molecules in plant responses to simultaneous stresses (i.e., high light and temperature)^30–32^ and to interact with phytohormones^30^. Both signal messengers play key roles during photooxidative and heat stress, leading to sun injury in apple fruit^3,29^. ABA, JA, SA, and ethylene have been reported to increase significantly during sun injury development on fruit attached to the tree^29^.

In Arabidopsis, cell wall damage has been associated with lignin accumulation mediated by ROS and JA^33^. JA and ethylene have been shown to be critical for modulating ROS levels in carrots subjected to abiotic stress^34^.

Lignin accumulation in green asparagus (*Asparagus officinalis* L.) postharvest has been shown to decrease (as well as ROS) when the asparagus is treated with 1-methylcyclopropene (1-MCP), GA_3_ or ethanol, and the opposite with exogenous ethylene treatment^35^. In poplar (*Populus trichocarpa*), the ethylene response factors (ERFs) are induced by ethylene, and most of them have been shown to be involved in wood development. In some cases, the overexpression of members of this class (i.e., ERF85 and ERF139) increased the amount of certain lignins^36^.

Together with the induction of lignin biosynthesis gene transcripts, photooxidative stress enhanced lignin deposition in the skin (Fig. 3). In the Fuji and Royal Gala, there was a significant accumulation of lignin after sudden exposure, particularly in sun-injured tissues (Fig. 3c, f). A significant accumulation of lignin was also observed in the flesh of sun-injured Fuji apples after 29 days (Fig. 3h). Nevertheless, this accumulation did not occur in Royal Gala, perhaps due to the shorter experiment duration in this cultivar (Fig. 3j). Certainly, these results indicate that the long-term exposure of apple fruit to abiotic stresses (high light and elevated temperature) in the field would increase lignin accumulation in cell walls^37^, leading to increased flesh firmness.

Lignification is a general mechanism that plants have developed to respond to damage caused by biotic (i.e., pathogens) and abiotic stresses, such as mechanical injuries, drought, and low/high temperatures^38,39^. It is also well known that sunlight affects lignin accumulation^39^ and that under normal conditions, lignin synthesis and deposition are affected by the photoperiod and light quality^40^.

In our study, lignin accumulation in apple fruit occurred as a result of photooxidative stress on the tree due to the upregulation of the phenylpropanoid pathway along with enhanced carbohydrate availability^7^. Both events are conducive to lignin biosynthesis. In fact, it has been proposed that the great flux of sugars from photosynthesis (i.e., glucose) in high light environments enhances the shikimic acid pathway to produce lignin^41,42^.

The activities of the enzymes PAL and CAD were increased by light in *Pinus radiata* seedlings when the seedlings were transferred from the dark to a 16 h photoperiod, resulting in an increase in their lignin content^43^. Moreover, Ali et al.^44^ reported similar results in orchids (*Phalaenopsis*) when leaves and roots were subjected to high light. The orchids accumulated more lignin, and their PAL, CAD and POD enzyme activities were higher.

However, heat stress also affects lignin accumulation by increasing the activity of laccase, which uses superoxide radicals to produce lignin^38^. This enzyme’s substrate, *O*-phenols, is readily available in apple tissue under high light conditions and most likely participates in the enzymatic browning that occurs during sun injury browning symptoms^1^. On the other hand, the burst in ROS that occurs during photooxidative stress in apples could also trigger lignin synthesis and deposition in cell walls^45^.

Increases in the lignin content have been well associated with higher fruit firmness in various fruit species. In strawberry, increased lignin was associated with enhanced firmness through the regulation of lignin biosynthesis^18^. The same authors proposed the *FaPOD27* gene, encoding a peroxidase involved in the later steps of lignin formation, as a candidate for improving fruit firmness. In loquat (*Eriobotrya japonica*), lignin accumulation is a negative event that occurs due to mechanical damage to the fruit, reducing its juiciness and consumer acceptance^17^. This phenomenon was shown to be associated with increased PAL, CAD and POD activities after 8 days at 20 °C^46^. In mangosteen (*Garcinia mangostana* L.), mechanical damage has also been shown to trigger a dramatic increase in lignin levels and pericarp hardening 30 min after the event^47^.

Ethylene is not only known as the ripening hormone in climacteric fruit, such as apples^48^, but also has important roles in plant development and abiotic stress defense^49^. Torres et al.^7^ reported higher levels of internal ethylene (nonripening related) early in the season in sun-injured apple tissue compared to noninjured apple tissue, suggesting its involvement in photooxidative and heat stress defenses. The expression analysis of *MdACS* and *MdACO* genes, which encode key enzymes in ethylene biosynthesis, showed a positive and consistent correlation with IEC only in the peel tissue of Sev Royal Gala fruit (*R* = 0.977; Table S2). In addition, skin *MdACS* was correlated well with IEC in the Exp category in both the Fuji and Royal Gala fruit (*R* = 0.928 and *R* = 0.686, respectively; Table S2). The relationship between this stress hormone and the upregulation of the lignin biosynthetic pathway has not been studied thus far, but ethylene has been shown to induce phenylpropanoid gene expression, specifically genes involved in flavonol synthesis, including *CHS*, *CHI* and *FLS*^50^. Our results indicate that IEC correlated well with *MdPAL* and *MdCHS* in both cultivars. In Fuji, the Sev skin and flesh tissues showed the highest correlations for *MdPAL* (*R* = 0.956 and *R* = 0.986, respectively; Table S2), there was a correlation only in the skin for *MdCHS* (*R* = 0.999; Table S2). In Royal Gala, only *MdCHS* in the skin and flesh of the Sev tissues was well correlated with IEC (*R* = 0.909 and *R* = 0.745, respectively; Table S2).

However, it has been reported that grape (*Vitis vinifera* L.) dehydration induces the expression of ethylene synthesis genes (ACO), along with genes related to phenylpropanoid metabolism^51^. In particular, flavonols and *trans*-resveratrol are induced, with a decrease in flavan-3-ols^52^. In addition, these authors found the upregulation of a laccase gene involved in lignin biosynthesis.

Considering that modifications in cell wall components are affected by environmental conditions and that lignification is highly regulated by light and elevated temperatures^38,39^, it is possible to postulate that an increase in the lignin content of apple flesh may explain, at least partially, the increase in fruit firmness, as previously suggested^37^. In conclusion, we showed that photooxidative stress caused by sudden sunlight exposure in apples induces lignin accumulation by the upregulation of phenylpropanoid genes and that ethylene is most likely involved in this defense response.

## Materials and methods

### Plant material and fruit sampling

Apple (*Malus* *×* *domestica* Borkh) trees from the Fuji and Royal Gala cultivars onto MM 106 rootstock grown in a commercial orchard in San Clemente, Chile (35°30'40” S., 71°28'26” W, 200 masl), were used in this study during the 2012–2013 growing season. The selected trees, which were 7 and 8 years old for Fuji and Royal Gala, respectively, were homogeneous in vigor and maintained with standard agronomic practices in a well-irrigated orchard. These cultivars were selected because they have different susceptibilities to sun damage, that is, the Fuji cultivar is highly susceptible, while the Royal Gala is highly tolerant, as well as different growing cycles. Royal Gala reaches horticultural maturity at approximately 120 DAFB, and Fuji reaches horticultural maturity at approximately 180 DAFB. Therefore, all fruit collections and sudden sun exposure experiments were carried out at different time points in each cultivar in the hottest period of the growing season (Fig. S1).

### Experiment 1

To evaluate changes in flesh firmness and the cell wall composition in response to sun exposure and sun injury, Fuji apples were picked at commercial harvest (180 days after full bloom, DAFB) and divided into different categories: nonexposed (Non Exp), shaded fruit sections unexposed to direct sunlight; exposed (Exp), fruit sections exposed to direct sunlight without sun injury symptoms; and severe (Sev), fruit sections with severe sun injury exhibiting both dark brown patches and discoloration on the fruit surface exposed to direct sunlight^29^. Three biological replicates were used per treatment or sun exposure category. Each replicate comprised tissue from 15 different fruit that were pooled and considered one biological replicate.

Fruit firmness was determined in each fruit section, after which the fruit flesh (parenchima tissue) was removed using a scalpel, flash frozen with liquid N_2_ and stored at −80 °C until further biochemical analyses.

### Experiment 2

Shaded fruit of the Fuji and Royal Gala cultivars were suddenly exposed to direct sunlight at 130 and 100 DAFB, respectively. Approximately 700 pieces of fruit were tagged on the tree and exposed to the sun manually by removing surrounding leaves. Tissue sampling (skin and flesh) was carried out after 0, 5, and 13 days for Gala fruit and 0, 4, 15, and 29 days for Fuji fruit. At each time-point, the fruit peel (exocarp and 5 layers of mesocarp) and flesh (parenchima) were removed using a scalpel, immediately frozen using liquid N_2_ and stored at −80 °C until use. A bulk tissue sample was prepared from each replicate.

Three biological replicates were used per treatment (sun exposure category × time point). Each replicate included tissue from 15 pieces of fruit.

The air temperature, solar radiation, and relative humidity values during these experiments in both cultivars are shown in Fig. S1. The selection of dates was based on the historic highest solar irradiation levels and daily maximum air temperatures during the summer in the southern hemisphere.

### Evaluations

#### Fruit firmness determination

Flesh firmness was measured at commercial harvest using 45 fruit (15 fruit per replicate) from each category (Non-Exp, Exp, Sev). This measurement was performed using a fruit texture analyzer with an 11-mm probe (GS14, GUSS Manufacturing, Strand, South Africa). Firmness was measured twice in each fruit section and the average recorded. The results were expressed in Newtons (N).

#### Internal ethylene concentration

The internal ethylene concentration (IEC) was determined by withdrawing 1 ml of air from the flesh of the fruit; air was immediately injected into a gas chromatography system (Hewlett Packard, Series II HP 5890, Agilent Technologies, Santa Clara, CA, United States) equipped with a flame ionization detector and a packed column (Porapak Q (80 °C oven)). The injector and detector temperatures were 150 °C and 200 °C, respectively. The gas flows for the N_2_ carrier, H_2_, and air were 25, 20, and 340 ml min^−1^, respectively. Five fruit per biological replicate (3 in total) were used for this determination.

Concentrations were determined using a standard curve. The results were expressed in parts per million (μL L^−1^) of ethylene^7^.

### Cell wall analysis

Cell wall material was obtained according to Vicente et al.^53^. In brief, 20 g of fresh tissue was homogenized in 80 mL of 100% ethanol and boiled for 30 min. The insoluble material was filtered and washed twice with 20 mL of boiling ethanol and 20 mL of 100% acetone. The alcohol-insoluble residue (AIR) was dried overnight at 37 °C and then weighed. The total content of cell wall material was expressed as g AIR per kg fresh weight (FW).

The fractionation of cell wall material was conducted by successive chemical treatment of AIR as previously reported by Vicente et al.^53^. In brief, 1 g of AIR was suspended in 100 mL of 0.02% (w/v) thimerosal solution and stirred for 16 h. The supernatant was filtered, and the pellet was washed twice with water. The filtrate and water washings were combined into the water-soluble fraction (WSF). The residue was then extracted with 20 mL of 50 mM trans-1,2-diaminocyclohexane-N,N,N′,N′-tetraacetic acid (CDTA) (pH 6.0) containing 0.02% (w/v) thimerosal for 12 h at room temperature. The mixture was centrifuged, and the pellet was washed twice with 15 mL of CDTA solution. The filtrate and washings were pooled into the CDTA-soluble fraction (CSF). The CDTA-insoluble pellet was then extracted with 20 mL of 50 mM Na_2_CO_3_ containing 20 mM NaBH_4_ for 30 min at 20 °C. The homogenate was filtered, and the pellet was washed twice with 20 mL of Na_2_CO_3_ containing NaBH_4_ solution and filtered for 40 min. The filtrate and washings were pooled into the Na_2_CO_3_-soluble fraction (NSF). The remaining pellet was extracted with 20 mL of 4 M KOH containing 20 mM NaBH_4_ for 1 h at 4 °C. The mixture was centrifuged, and the pellet was washed twice with 15 ml of KOH solution. The filtrate and washings were neutralized with acetic acid, becoming the KOH-soluble fraction (KSF) equivalent to the hemicellulose fraction. Finally, the remaining pellet was washed with 50 mL of 50% acetic acid, and its residue was washed with 50 mL of ethanol:diethyl ether (1:1) and dried. The remainder was dissolved in 0.6 mL H_2_SO_4_ 72% and later diluted in water and heated at 120 °C to become the sulfuric soluble fraction (SSF) equivalent to the cellulose fraction. Three independent extractions were performed for each replicate.

### Uronic acid (UA) and neutral sugar (NS) measurements

The concentrations of UA and NS in the obtained cell wall fractions were determined colorimetrically as previously reported^54,55^. The results were calculated using a standard curve of galacturonic acid (UA) or glucose (NS). All measurements were performed in triplicate.

### Total RNA extraction and cDNA synthesis

Total RNA extractions were performed based on the CTAB method described by Chang et al.^56^ and adapted to apple^57^. Three grams of peel and 9 g of flesh of apple fruit bulk were used for each extraction replicate. Contaminant DNA was removed from the RNA samples using RNase-free DNase I treatment (Promega, Madison, WI, USA) according to the manufacturer’s instructions. RNA integrity was evaluated using 1% agarose gel electrophoresis, whereas the RNA concentration and purity (OD260/OD280 ratio >1.95) were assessed by optical density in a NanoDrop ND-1000 spectrophotometer (NanoDrop Technologies, Montchanin, DE, USA). Three independent RNA extractions were conducted for each sample. First strand cDNA synthesis was performed using the First Strand cDNA Synthesis Kit (Fermentas Life Science, Glen Burnie, MD, USA) following the manufacturer’s instructions.

### Quantitative real-time PCR

Gene transcript levels were analyzed by quantitative PCR (qRT-PCR). Phenylpropanoid-related genes were selected based on the apple transcriptomes and genome sequences available from http://compbio.dfci.harvard.edu/tgi/. Selected orthologous genes were those whose transcripts were only expressed in fruit development stages and not in other vegetative tissues. Primers for qPCR were designed using Beacon Designer v 2.0 software (PremierBiosoft, Palo Alto, CA, USA)^58^. The primers used for qPCR analysis are shown in supplementary Table S1. Serial dilutions of amplified PCR products were used as standard templates to assess the PCR efficiency for each primer pair (Table S1). Experiments were performed using three biological replicates, and each PCR run was carried out with three technical replicates. The protocol used was reported in Ramos et al.^59^. Data were analyzed using the Excel (Microsoft) macro GENEX v1.10 (gene expression analysis for iCycle iQ® real-time PCR detection system, v1.10, 2004; Bio-Rad Laboratories) using methods derived from the algorithm of Vandesompele et al.^60^. Gene expression data were obtained from three individual experiments (biological and technical replicates) and were analyzed using a two-way ANOVA-LSD post hoc test, and significant differences were determined at *P* ≤ 0.05. Transcript levels (mRNA abundance) of genes involved in the phenylpropanoid pathway in apple were obtained from three individual experiments (biological and technical replicates) and normalized against *EIF4a* (AY347787) and *EF-1*(XM_008367439)^10^.

### Lignin determination

Lignin was extracted following the protocol previously described by Campbell and Ellis^61^. Two hundred milligrams of ground skin or flesh tissue were mixed with 1.5 ml of 0.1 M phosphate buffer (pH 7.2). Samples were diluted with 1 M NaOH (1:3, v/v) and then hydrolyzed. Briefly, each sample was mixed with 750 µl of distilled water, 250 µl HCl (37%) and 100 µl of thioglycolic acid (Sigma Aldrich, St. Louis, MO, USA) and then incubated at 80 °C for 3 h. Lignin absorbance was determined spectrophotometrically at 280 nm. The results were expressed as µg lignin per kg of FW.

### Histological preparations

Sample preparation was performed based on Siegel^62^ with modifications based on Ramos and Herrera^63^. Briefly, fresh tissues were cut and fixed at 4 °C in fresh fixative solution composed of 4% (v/v) formaldehyde, 50 mM 1,4-piperazinediethanesulfonic acid (PIPES), 5 mM MgSO_4_, 5 mM EGTA, 0.01% (v/v) Triton X-100, and 0.1% (v/v) dimethyl sulfoxide (DMSO), with a pH of 6.9, for at least 48 h. Then, samples were dehydrated by sucrose solutions (10, 20, 30, and 40%). Samples were transversely sectioned (30 µm thick) using an ice sliding microtome (Leica CM1325, Leica Microsystems, Wetzlar, Germany). In order to indicate the lignin deposition in the cell walls of the skin and flesh cells, fruit sections were stained with phloroglucinol-HCl and viewed under a light microscope^62^.

### Statistical analysis

Data were analyzed using analysis of variance, and mean differences were separated using Tukey´s test (*P* ≤ 0.05) when ANOVA *P* ≤ 0.05. Pearson correlation coefficients were calculated to assess relationships between IEC and gene expression. Statistical procedures were performed using SAS (version 8.02) software.

## Supplementary information

Supplementary information
