# Supplementary material for: Photooxidative stress activates a complex multigenic response integrating the phenylpropanoid pathway and ethylene, leading to lignin accumulation in apple (Malus domestica Borkh.) fruit
Source: Hortic Res. 2020 Mar 1;7:22. doi: 10.1038/s41438-020-0244-1 (PMC7049307; doi:10.1038/s41438-020-0244-1)
Supplement: Supplementary file 1 — Supplementary information [file 41438_2020_244_MOESM1_ESM.docx]

**
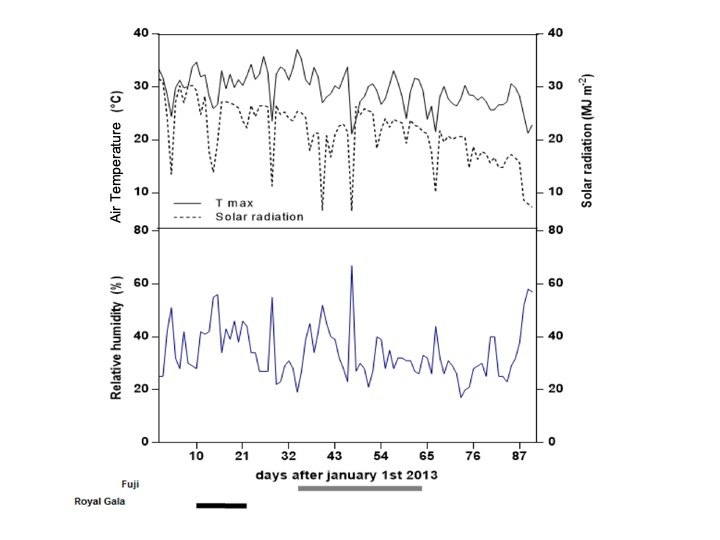
**

**Figure S1.** Daily maximum air temperature (°C), solar radiation (MJ m^-2^) and relative humidity (%) during the 2013 growing season (days after January 1^st^) in San Clemente, Chile (35°30'40" S., 71°28'26" W). The gray and black bars at the bottom of the figure represent the time-course experiment after sudden exposure to sunlight in Fuji and Royal Gala apples, respectively.

**Table S1**. List of primers used in RT-qPCR.

**Table S2**. Pearson coefficients for IEC vs *MdPAL*, *MdCHS*, *MdF3*H, *MdCOMT1*, *MdCAD*, *MdACS,* and *MdACO* expression.

| Cultivar | Tissue |  | *MdPAL* | *MdCHS* | *MdF3*H | *MdCOMT1* | *MdCAD* |
| --- | --- | --- | --- | --- | --- | --- | --- |
| Fuji | Skin | Non-Exp | -0.484 | 0.671 | 0.878 | 0.975 | 0.994 |
|  |  | Exp | 0.820 | 0.425 | 1.000 | -0.356 | 0.457 |
|  |  | Sev | 0.956 | 0.999 | 0.995 | 0.827 | -0.734 |
|  | Flesh | Non-Exp | -0.875 | -0.989 | -0.699 | 0.998 | 0.935 |
|  |  | Exp | -0.628 | 0.904 | 0.035 | -0.367 | 0.456 |
|  |  | Sev | 0.986 | 0.153 | 0.321 | 0.731 | 0.790 |
| Royal Gala | Skin | Non-Exp | 0.702 | 0.412 | 0.759 | 0.159 | 0.143 |
|  |  | Exp | 0.624 | 0.717 | 0.986 | 0.553 | 0.524 |
|  |  | Sev | 0.178 | 0.909 | -0.485 | -0.279 | -0.732 |
|  | Flesh | Non-Exp | -0.917 | -0.252 | -0.062 | -0.566 | -0.965 |
|  |  | Exp | 0.984 | 0.153 | -0.100 | 0.181 | 0.225 |
|  |  | Sev | -0.213 | 0.745 | 0.225 | 0.754 | 0.442 |
